# Les manifestations oculaires au cours de la pré-éclampsie sévère ou l’éclampsie au Centre Hospitalier Universitaire Sourô Sanou de Bobo Dioulasso

**DOI:** 10.11604/pamj.2015.21.49.6746

**Published:** 2015-05-25

**Authors:** Jean Wenceslas Diallo, Nonfounikoun Méda, Ahgbatouhabéba Ahnoux-Zabsonré, Souleymane Ouattara, Armande Yanogo, Somnoma Jean Baptiste Tougouma, Der Somé, Jérôme Sanou, Mariam Dolo

**Affiliations:** 1 Institut Supérieur des Sciences de la Santé (INSSA), Université Polytechnique de Bobo Dioulasso (UPB), Centre Hospitalier Universitaire Sourô Sanou (CHUSS); 2 Unité de Formation et de Recherche en Sciences de la Santé, Université de Ouagadougou, Centre Hospitalier Universitaire Yalgado Ouédraogo

**Keywords:** Pré-éclampsie, éclampsie, manifestations oculaires, Burkina Faso, Pre-eclampsia, eclampsie, ocular manifestations, Burkina Faso

## Abstract

La pré-éclampsie sévère est un problème de santé publique. L'atteinte oculaire est une de ses nombreuses complications. Le but de notre travail était de décrire les atteintes oculaires chez les patientes présentant une pré-éclampsie et/ou éclampsie afin de contribuer à leur meilleure prise en charge. Il s'est agi d'une étude transversale descriptive à collecte prospective allant du 1^er^ novembre 2013 au 31 juillet 2014, chez les patientes ayant souffert de pré-éclampsie sévère/éclampsie. Nous avons inclus 127 patientes dans notre étude. La moyenne d’âge des patientes de notre étude était de 26,37 ans (ET= 6,8 ans), avec des extrêmes de 15 et 40 ans. Les tranches d’âge les plus représentées étaient celles de 26 à 30 ans avec 29,1% des cas et celle des 15 à 20 ans avec 25,2%. Le diagnostic de pré-éclampsie sévère a été retenu dans 69,3% des cas. Les primigestes représentaient 40,9% de la population. Les troubles visuels à type de phosphènes ont été observés chez 33,1% des patientes. Nous avons noté un courant granulaire conjonctival dans 41,7%, des lésions du segment postérieur chez 32,3% des patientes. Ces résultats ont été discutés par rapport à la littérature, et nous notons plus de cas d'atteinte rétinienne. Nous n'avons pas trouvé de lien statistiquement significatif entre la tension artérielle à l'admission et le stade de la rétinopathie hypertensive. Les complications oculaires de la pré-éclampsie sévère sont très fréquentes et souvent ignorées. Les atteintes rétiniennes sont fréquentes mais de bon pronostic.

## Introduction

La Pré-éclampsie est définie parl'Organisation Mondiale de la Santé (OMS) comme étant l'association d'une hypertension artérielle, d'une protéinurie supérieure à 300mg/24 heures ou supérieure à 2 croix à la bandelette urinaire à partir de la 20^ème^ semaine d'aménorrhée avec ou sans œdèmes des membres inférieurs [1]. La pré-éclampsie sévère (PES) est définie par l'apparition à partir de la 20^ème^ semaine d'aménorrhée d'une hypertension artérielle dont la tension artérielle systolique (TAS) est supérieure ou égale à 160 mmHg et/ou la tension artérielle diastolique (TAD) supérieure ou égale à 110 mmHg, et d'une protéinurie supérieure ou égale à 3g/24heures ou supérieure ou égale à 3 croix à la bandelette urinaire [1]. La PES peut se compliquer d'une éclampsie qui est un accident paroxystique à expression neurologique dominante, se manifestant par des crises convulsives tonico-cloniques survenant dans un contexte de pré-éclampsie sévère méconnue ou non traitée. Elle réalise un état convulsif à répétition suivi d'un état comateux et peut survenir aussi bien au cours de la grossesse après la 20^ème^ semaine d'aménorrhée, pendant l'accouchement, ou dans le post-partum [1]. La pré-éclampsie/éclampsie est un problème de santé publique. En effet elle constitue l'une des 3 premières causes de mortalité maternelle dans le monde [1, 2]. Sa prévalence est de 25% des femmes enceintes en Afrique subsaharienne et elle figure parmi les 4 premières causes de décès maternels au Burkina Faso [3, 4]. Les atteintes oculaires concernent 25% des femmes atteintes de PES et jusqu’à 50% des femmes atteintes d’éclampsie [5]. Les complications oculaires de la pré-éclampsie sont essentiellement la rétinopathie hypertensive, le décollement de la rétine, l'hémorragie du vitrée. Au Burkina Faso très peu d’études ont été menées sur le sujet et nous disposons de peu d'informations sur les complications oculaires dues à la pré-éclampsie/éclampsie. Le but de notre travail a été d’étudier les atteintes oculaires en particuliers les aspects du fond d’œil chez les femmes atteintes de PES ou d’éclampsie au Centre Hospitalier Universitaire Sourô Sanou (CHUSS), afin de contribuer à leur meilleure prise en charge.

## Méthodes

Sur le plan éthique, nous avons requis le consentement éclairé signé des patientes et la confidentialité pour chacune a été respectée. Le refus de la patiente n'entraînait aucune incidence sur sa prise en charge. Le cadre de notre étude a été la ville de Bobo Dioulasso, 2^ème^ ville du Burkina Faso, et le champ le Centre Hospitalier Universitaire Sourô Sanou (CHUSS) qui est le dernier niveau de référence dans la région sud-ouest du pays. Nous avons mené une étude transversale descriptive à collecte prospective qui s'est déroulée du 1^er^ novembre 2013 au 31 juillet 2014. La population d’étude était constituée des femmes ayant accouché dans un contexte de PES ou d’éclampsie diagnostiquée et/ou traitée au CHUSS. Ont été incluses dans notre étude les femmes enceintes ayant souffert de pré éclampsie sévère/éclampsie qui ont accouché et/ou ont été reçues et traitées au CHUSS entre le 1^er^ novembre 2013 et le 31 juillet 2014 et qui ont donné leur consentement éclairé par écrit. Les critères de non inclusion étaient les femmes qui n'ont pas donné leur consentement éclairé, ou les femmes souffrant d'hypertension artérielle avant le début de la grossesse. Nous avons effectué un échantillonnage aléatoire simple. Toutes les patientes reçues en ophtalmologie au cours de la période et répondant aux critères d'inclusion ont été retenues pour notre étude. Nous avons relevé les variables en rapport avec les données sociodémographiques, le déroulement de la grossesse, les antécédents, les données de l'examen clinique général et de l'examen ophtalmologique. Dans certains cas une angiographie à la fluorescéine a été pratiquée. Nous avons adopté les définitions opérationnelles suivantes: **une protéinurie significative**: la protéinurie est considérée comme significative lorsqu'elle est supérieure ou égale à 2 croix à la bandelette urinaire; **la gestité**: les patientes sont dites primigestes lorsqu'il s'agit de la première grossesse, paucigestes lorsqu'elles ont eu 2 à 4 grossesses, multigestes entre 5 et 7 grossesses, et grandes multigestes au-delà de 7 grossesses; **la parité**: les patientes sont dites paucipares lorsqu'elles ont entre 1 et 4 accouchements, multipares lorsqu'elles ont eu 5 à 7 accouchements, grandes multipares au-delà de 7 accouchements; **consultation pré natale**: nous considèrerons comme acceptable un nombre de consultations pré natales (CPN) supérieur ou égale à 3 durant la grossesse; **patiente référée**: patiente adressée par un centre de santé vers un autre centre de santé d’échelon supérieur pour une meilleure prise en charge en dehors de toute situation d'urgence; **patiente évacuée**: patiente orientée d'urgence par un centre de santé vers un autre centre de santé d’échelon supérieur et transportée dans le dit centre par une ambulance; **patiente venue d'elle-même**: patiente s’étant présentée directement au CHUSS sans être passée par un autre centre de soins. Les informations sur nos patientes ont été recueillies par entretien individuel grâce à un questionnaire préalablement validé par un pré-test. Les données ont été saisies et analysées par le logiciel SPSS dans sa version 20. Les tableaux et graphiques ont été réalisés à l'aide du logiciel EXCEL 2010. Pour les variables quantitatives indépendantes, les moyennes accompagnées des écarts types ont été utilisées, pour les variables qualitatives, nous avons utilisé les fréquences relatives. Le test de Chi2 de Pearson a été utilisé pour la comparaison des proportions. Le seuil de signification pour tous les tests statistiques a été fixé à 5%.

## Résultats

Au cours de la période d’étude de novembre 2013 à juillet 2014, 3398 accouchements ont été enregistrés au CHUSS dont 590 ont présenté une pré-éclampsie sévère ou une éclampsie soit 17,36%. Parmi ces dernières, 131 patientes ont eu un examen du fond d’œil soit 22,20%, dont 127 répondaient aux critères d'inclusion. La pré-éclampsie sévère était le diagnostic le plus fréquent parmi elles avec 69,3% (n=88) des cas, 28 patientes soit 22% présentaient une éclampsie, et 11 (8,7%) un état de mal éclamptique. La majorité de nos patientes résidait dans la ville de Bobo Dioulasso avec 77,2% (n=98).

### Caractéristiques sociodémographiques

**Figure 1:**
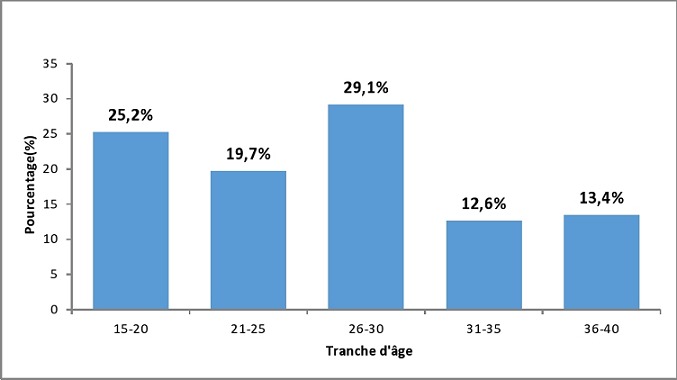
Répartition des 127 patientes selon les tranches d’âge

La moyenne d’âge des 127 patientes était de 26,37 ans (ET= 6,8 ans), avec des extrêmes de 15 et 40 ans (Figure 1). Les tranches d’âge les plus représentées étaient celles de 26 à 30 ans avec 37 cas soit 29,1% et celle des 15 à 20 ans avec 32 cas soit 25,2%. Dans notre série 48,8% des patientes n’étaient pas scolarisées. Les patientes ayant le niveau primaire ou secondaire représentaient 23,6% (n=30) des cas pour chaque groupe. Les femmes au foyer représentaient 63,8% (n=81) des cas. Les femmes mariées constituent la majorité de la population d’étude avec 96 cas soit 75,6%.

### Antécédents

Les antécédents sont récapitulés dans le Tableau 1. Nous avons noté autant de patientes primigestes 40,9% (n=52), que de paucigestes 40,2% (n=51). Les patientes paucipares représentaient 85% (n=108) des cas. Les antécédents de pathologies générales ont été retrouvés dans 4 cas soit 3,2%, et la pathologie la plus fréquente était la drépanocytose avec 1,6% (n=2) des cas. Six patientes avaient déjà souffert de PES ou d’éclampsie aux grossesses antérieures soit 4,7%. Trois patientes soit 2,4% avaient déjà subi une césarienne, dont une pour PES. Nous n'avons pas noté d'antécédent ophtalmologique en dehors de 3 patientes qui avaient une amétropie corrigée par des lunettes.

**Tableau 1:** Répartition des 127 patientes selon les antécédents

Antécédents	Effectif	Pourcentage
**Gestité**		
Primigeste	52	40,9
Paucigeste	51	40,2
Multigeste	19	15
Grande gestité	5	3,9
**Parité**		
Paucipare	108	85
Multipare	15	12
Grande parité	4	3
**Pathologie générale**		
Diabète	1	0,8
Drépanocytose	2	1,16
Asthme	1	0,8

### Déroulement de la grossesse et accouchement

La plupart des patientes a consulté régulièrement pour le suivi de la grossesse. En effet, 101 patientes soit 79,5% ont été suivies pendant la grossesse par 3 CPN au moins. Le mode d'admission au CHUSS le plus fréquemment rencontré était la référence qui représentait 34,6% (n=44) des cas. La majorité de nos patientes avait une grossesse à terme au moment de l'accouchement avec 75,6% (n=96) des cas. L'accouchement a été majoritairement par voie basse 75,6% des cas (n=96), et a donné naissance à un fœtus dans 90,6% des cas (n=115) et 2 fœtus dans 9,4% des cas (n=12). Le nouveau-né était vivant dans 82,7% des cas (n=105).

### Signes cliniques à l'admission

Les signes cliniques à l'admission sont récapitulés dans le Tableau 2. L’état de conscience des patientes était bon dans 71,7% des cas (n=91). Parmi les patientes, 33,1% (n=42) ont présenté un trouble visuel dont 19,7% (n=25) à type de phosphènes. Lors de l'admission 62,2% (n=79) des patientes avaient une tension artérielle systolique supérieure ou égale à 160 mmHg avec un extrême à 230mmHg. La tension artérielle diastolique était supérieure ou égale à 110mmHg dans 51,2% (n=65) avec un extrême à 150mmHg. Les œdèmes des membres inférieurs ont été notés dans 76,4% (n=97) des cas et 72,4% (n=92) des patientes ont présenté des céphalées pendant le travail d'accouchement et/ou après l'accouchement. La protéinurie à la bandelette urinaire lors de l'admission était de 4 croix dans 48,8% (n=62) de 3 croix dans 41,7% (n=53) des cas. Les complications de la PES ont été observées chez 7,9% (n=10) des patientes. Nous avons noté 4 cas (3,1%) d'hématome retro-placentaire. Les complications à type d’œdème aigu du poumon, d’état de mal éclamptique et d’éclampsie ont été observées chacune chez 2 (1,6%) patientes. Un cas de HELLP syndrome (0,8%) a été noté.

**Tableau 2:** Répartition des 127 patientes selon les signes cliniques à l'admission

Signes cliniques	Effectif	Pourcentage
**Etat de conscience à l'entrée**		
Bon	91	71,7
Altéré	25	19,7
Coma	11	5,6
**Protéinurie à la bandelette urinaire**		
2 croix	12	9,4
3 croix	53	41,7
4 croix	62	48,9
**Tension artérielle**		
**TAS à l'entrée**		
140 ≤ TAS < 160 mmHg	48	37,8
TAS ≥ 160 mmHg	79	62,2
**TAD à l'entrée**		
90 ≤ TAD < 110 mmHg	62	48,8
TAD ≥ 110 mmHg	65	51,2
**Œdème des membres inférieurs**		
Oui	97	76,4
Non	30	23,6
**Troubles visuels**		
Aucun	85	66,9
Flou visuel	10	7,9
Amaurose subite	7	5,5
Phosphène	25	19,7

### Données de l'examen ophtalmologique

**Figure 2:**
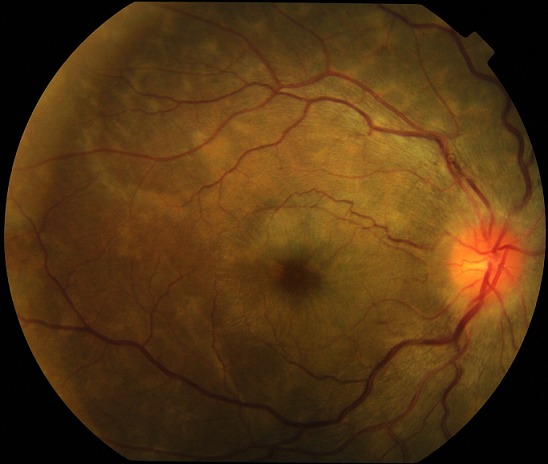
Rétinographie d'un œil droit: rétrécissement diffus des artères, dilatation veineuse, tortuosité vasculaire, décollement séreux rétinien avec des plis rétiniens maculaires, et lésions multiples d'ischémie choroïdienne. Acuité visuelle: 2/10

**Figure 3:**
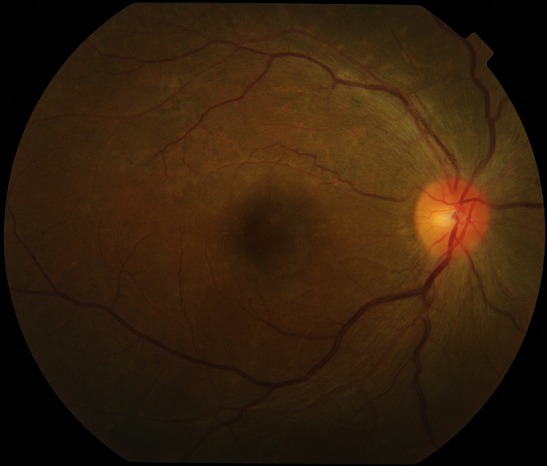
Rétinographie de la même patiente que la figure 1: évolution favorable après 1 mois avec réversion de la tortuosité vasculaire et de la dilatation veineuse, assèchement du décollement séreux rétinien. Acuité visuelle: 10/10

Le Tableau 3 montre les données de l'examen ophtalmologique. La grande majorité des patientes 97,6% (n=124) présentait une acuité visuelle supérieure ou égale à 3/10. Nous avons noté 2 cas de cécité transitoire avec une acuité visuelle post critique inférieure à 1/20. L'acuité visuelle de contrôle à J60 réalisée chez 20 patientes ne notait pas de déficit visuel. L'examen de la conjonctive était le plus souvent normal, l'anomalie la plus fréquente était la présence d'un courant granulaire au niveau des 2 yeux dans 41,7% (n=53) des cas. La cornée était sans anomalie chez toutes les patientes ainsi que la pression intra oculaire. Au niveau du segment postérieur, nous avons noté des lésions chez 32,3% (n=41) des patientes dont les plus fréquentes étaient la tortuosité vasculaire 90,2% (n=37), le rétrécissement artériel diffus 75,6% (n=31), et les nodules cotonneux 73,1% (n=30). Les lésions observées au fond œil ont été presque toujours bilatérales et symétriques dans 98,4% (n=125) des cas (Figure 2). La rétinopathie hypertensive était présente chez 26% (n=33) des femmes ayant souffert de PES et/ou d’éclampsie. L'ischémie choroïdienne et le décollement de rétine exsudatif représentaient respectivement 14,6% et 4,8% des lésions rétiniennes. Ces atteintes étaient accompagnées d'une baisse de l'acuité visuelle avec une évolution rapidement favorable (Figure 3). Les rétinopathies hypertensives stade 1 et stade 2 de Kirkendall ont été les plus représentées avec respectivement 12,6% (n=16) et 9,4% (n=12) des cas pour les yeux droits, 12,6% (n=16) et 11% (n=14) des cas pour les yeux gauches lors de l'examen initial. Parmi les 41 patientes ayant présenté des anomalies du fond œil seulement 48,8% (n=20) ont été revues au contrôle du 60^ème^ jour. Le fond d’œil de contrôle était normal dans 90% (n=18). Nous n'avons pas retrouvé de lien statistiquement significatif entre la tension artérielle systolique à l'entrée et le stade de rétinopathie hypertensive observée p=0,31, ni entre la tension artérielle diastolique à l'entrée et le stade de la rétinopathie hypertensive p=0,36.

**Tableau 3:** Répartition des patientes selon les résultats de l'examen ophtalmologique. (n= dénominateur pour la variable concernée)

Signes cliniques	Effectif	Pourcentage
**Acuité visuelle (AV) à l'admission (n=127)**		
Pas de déficience visuelle: AV ≥ 3/10	124	97,6
Déficience visuelle: 3/10 < AV < 1/20	1	0,8
Cécité: AV < 1/20	2	1,16
**Acuité visuelle au contrôle (n=20)**		
Pas de déficience visuelle: AV ≥ 3/10	20	100
**Courant granulaire conjonctival (n=127)**	53	41,7
**Lésions rétiniennes (n=127)**	41	32,28
**Type de lésions rétiniennes (n=41)**		
Tortuosité vasculaire	37	90,2
Rétrécissement artériel diffus	31	75,6
Dilatation veineuse	22	53,6
Hémorragie rétinienne	20	48,7
Hémorragie pré rétinienne	0	0
Nodules cotonneux	30	73,1
Œdème papillaire	11	26,8
Ischémie choroïdienne	6	14,6
Décollement séreux neuro rétinien	2	4,8
Œdème maculaire	1	2,4
Décollement de rétine exsudatif	2	4,8
Occlusion vasculaire	0	0

## Discussion

### Les limites de l’étude

Au cours de la période de l’étude la fréquence de la PES et l’éclampsie parmi les femmes qui ont accouché au CHUSS a été de 17,36% (n=590). Parmi ces dernières, moins d'un quart 22,2% soit 127 patientes ont été vues en ophtalmologie et ont constituées notre population d’étude. Cette faible proportion pourrait entraîner un biais dans nos résultats comparé à un échantillon plus grand. La méconnaissance des retentissements oculaires de la maladie par le personnel soignant et par les patientes pourrait expliquer en partie ce faible taux. Une autre limite a été la faible compliance des patientes au contrôle. En effet, seulement la moitié de celles qui présentaient des anomalies du fond d’œil sont revenues à l'examen de contrôle. Ceci pourrait également impacter nos statistiques. Toutefois, nous pensons que le caractère prospectif de la collecte des données, et l'aspect innovant de ce travail constituent les points forts de notre étude. Nous discuterons les caractéristiques sociodémographiques, et les données cliniques en particulier les aspects ophtalmologiques.

### Caractéristiques sociodémographiques

La moyenne d’âge était de 26,37 ans (ET= 6,8 ans), avec des extrêmes de 15 et 40 ans. Nos résultats sont comparables à ceux de Ouédraogo au Burkina Faso et de Bah en Guineé qui trouvaient dans leurs travaux respectivement 25,5 ans et 25ans [3, 4]. Dans la littérature cette moyenne varie de 22 à 28 ans [6, 7]. Ces différences dans les moyennes d’âges pourraient s'expliquer par les caractéristiques sociales et cultuelles des populations étudiées, ainsi que leur niveau d'instruction. Dans notre série près de la moitié des patientes soit 48,8% n’étaient pas scolarisées, ceci est comparable aux résultats de Fomba à Bamako qui trouvait 51,8% [8]. Des taux plus élevés ont été rapportés par Ouédraogo à Ouagadougou 57,2% et Bah en Guinée 73,47% [3, 4]. Les femmes au foyer étaient majoritaires avec 63,8% des cas comme cela est souvent rapporté dans la littérature en Afrique de l'ouest [3, 8, 9].

### Données cliniques

Les primipares représentaient 40,9% (n=52) des patientes dans notre étude, et les paucipares 85%, alors que Fomba en rapporte 28% de ses cas au Mali [8]. La primiparité est connue pour être un facteur de risque de la pré-éclampsie et sa proportion est variable dans la littérature [10, 11]. A l'admission, la PES était le diagnostic le plus fréquent 69,3% (n=88) des cas dans notre étude, alors que Fomba trouvait un résultat inférieur avec 51,8% des cas [8]. La tension artérielle élevée est un signe de gravité de la pré-éclampsie. Nous rapportons 62,2% des TAS supérieure ou égale à 160mmHg résultat comparable à celui de Samaké qui trouvait 58,46% [9]. A l'examen ophtalmologique, le signe fonctionnel prédominant a été un trouble visuel avec 33,1% (n=42) des patientes dont 19,7% (n=25) à type de phosphènes, alors que Samra trouvait une valeur inférieure avec 25% des cas [5]. Nous avons noté 2 cas de cécité transitoire avec une acuité visuelle post critique inférieure à 1/20 d’évolution favorable. Cunningham rapporte 15 cas de cécité transitoire de quelques heures à 8 jours, d'origine corticale, secondaire à une hémorragie et/ou un œdème focal du cortex occipital d’évolution favorable [12]. Chez 41,7% des patientes nous avons observé un courant granulaire au niveau de la conjonctive, comme rapporté par Omoti [13] et serait en rapport avec une légère hémodilution. Dans notre échantillon 32,3% des patientes présentaient des lésions rétiniennes, supérieur au taux rapporté par Karki qui trouvait 13,7% [14]. Cette grande différence pourrait s'expliquer par les outils utilisés pour l'examen du fond d’œil. Les lésions rétiniennes étaient dominées par les tortuosités vasculaires 90,2% et le rétrécissement diffus des artères 75,6% proche des 70% rapporté par Samra et d'autres auteurs [5, 15, 16]. La rétinopathie hypertensive était présente chez 26% (n=33) des patientes. Tadin trouvait des résultats supérieurs avec 45% des cas [17]. Cette différence pourrait s'expliquer par la méthodologie. En effet nous avons utilisé la classification de Kirkendall de la rétinopathie hypertensive, alors que Tadin a utilisé la classification de Keith et Wegener. Cette dernière a l'inconvénient de traiter à la fois des signes dus à l'hypertension artérielle et à l'artériosclérose. Nous n'avons pas retrouvé de lien statistiquement significatif entre la tension artérielle systolique à l'entrée et le stade de rétinopathie hypertensive observée p=0,31, ni entre la tension artérielle diastolique à l'entrée et le stade de la rétinopathie hypertensive p=0,36. Cette observation a été également faite par Moshiri qui indique que la sévérité de la rétinopathie hypertensive n'est pas corrélée à la TAS ou à la TAD [18]. Gupta ne trouvait pas non plus de lien statistique entre la sévérité de la PES et l'atteinte oculaire [19]. Selon ce dernier, la sévérité de la rétinopathie hypertensive serait plutôt liée au niveau d'insuffisance placentaire et non à la tension artérielle.

## Conclusion

La pré éclampsie/éclampsie constitue un problème de santé publique. Elle affecte près d'une femme sur cinq qui vient accoucher au CHUSS. Moins d'un quart de ces femmes ont été vues en ophtalmologie alors que les complications rétiniennes ont été notées dans plus d'un tiers des cas. Afin de mieux prendre en charge ces patientes, il serait pertinent de mener des actions de sensibilisation à l'endroit du personnel médical et des patientes. Des études ultérieures seraient utiles pour explorer davantage les mécanismes des diverses atteintes rétiniennes.
